# Hybrid blood purification strategy in pediatric septic shock

**DOI:** 10.1186/s13054-016-1535-7

**Published:** 2016-11-10

**Authors:** Gabriella Bottari, Fabio Silvio Taccone, Andrea Moscatelli

**Affiliations:** 1 Istituto Giannina Gaslini, Neonatal and Paediatric Intensive Care Unit, Largo Gerolamo Gaslini 5, 16147 Genoa, Italy; 2 Department of Intensive Care, Hopital Erasme, Route de Lennik 808, 1070 Brussels, Belgium

Extracorporeal blood purification techniques (EBTs) are emerging as beneficial interventions in the management of sepsis. Although the combination of different EBTs has also been suggested as a potentially effective approach in the early phases of sepsis [1, 2], no data are available for pediatric septic shock.

We reported a case of refractory septic shock in a 12-year-old girl, with a clinical history of acute lymphatic leukemia and recent chemotherapy, who was admitted to the emergency department (ED) because of fever and fatigue. In the ED ward, mean arterial pressure (MAP) was below 50 mmHg and did not improve after initial fluid resuscitation (20 ml/kg). Empiric antimicrobial therapy was initiated and the patient was admitted to the pediatric intensive care unit. Because of severe persistent hypotension with arterial lactate concentrations of 74 mg/dl, epinephrine and norepinephrine were initiated at 0.2 μg/kg/min and 0.08 μg/kg/min, respectively. Six hours after admission, the patient remained severely hypotensive (MAP of 45 mmHg) despite fluid and vasopressor therapy and low-dose hydrocortisone administration. In the absence of oliguria, continuous renal replacement therapy (CRRT) was started (continuous venovenous hemodiafiltration modality; effluent of 35–40 ml/kg.h) with a high cutoff (HCO) filter (Septex®) in combination with a cartridge column (CC; Cytosorb®). A significant reduction of vasopressor doses was observed 48 hours after the initiation of EBT (Fig. 1). A similar positive trend was observed for lactate (74 vs 32 mg/dl) and procalcitonin (65 vs 18 ng/ml) concentrations. This “hybrid” EBT was continued for 72 hours without adverse events. The patient was successfully discharged after 10 days. The blood cultures yielded *Klebsiella pneumonia*, related to a percutaneously inserted central line infection.

**Fig. 1:**
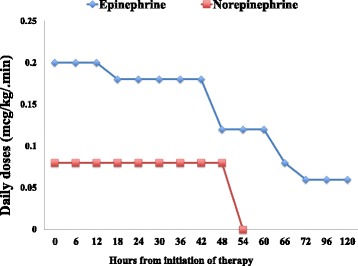
Vasopressor and inotrope infusion rates during extracorporeal blood purification (EBT)

In this case, the benefits of CRRT in the management of fluid overload and metabolic abnormalities in children with septic shock were associated with the immune-modulatory effects of EBTs. HCO filters are characterized by large pore size (e.g., cutoff value of 70 kDa) with improved convective clearance of middle molecular weight molecules, such as cytokines [3]. CCs have a large surface area that could also directly adsorb and clear inflammatory mediators [4]. The combination of HCO-CRRT and CC might have a synergistic effect in this setting [5]; this association has not yet been explored in the treatment of pediatric septic shock. Further studies are needed to assess the feasibility as well as the optimal timing of initiation of such an approach in children suffering from septic shock.

## Abbreviations

CC: Cartridge column
CRRT: Continuous renal replacement therapy
EBT: Extracorporeal blood purification technique
HCO: High cutoff
MAP: Mean arterial pressure

## References

[1] Zhou F, Peng Z, Murugan R, Kellum JA (2013). Blood purification and mortality in sepsis: a meta-analysis of randomized trials. Crit Care Med.

[2] Kellum JA, Gómez H, Gómez A, Murray P, Ronco C, ADQI XIV WORKGROUP (2016). Acute dialysis quality initiative (adqi) xiv sepsis phenotypes and targets for blood purification in sepsis: the bogotá consensus. Shock.

[3] Morgera S, Slowinski T, Melzer C (2004). Renal replacement therapy with high cutoff hemofilters: impact of convenction and diffusion on cytokine clearance and protein status. Am J Kidney Dis.

[4] Kellum JA, Song M, Venkataraman R (2004). Hemoadsorption removes tumor necrosis factor, interleukin-6, and interleukin-10, reduces nuclear factor-kB DNA binding, and improves short-term survival in lethal endotoxemia. Crit Care Med.

[5] Honore P, Jacobs R, Joannes-Boyau O (2013). Newly designed CRRT membranes for sepsis and SIRS-a pragmatic approach for beduine intensivists summarizing the more recent advances: a systematic structured review. ASAJO J.

